# Medicine in the Great War

**Published:** 1917-11-10

**Authors:** 


### The Hospital

The Workers' Newspaper of Administrative Medicine and Institutional Life, Administration, National Insurance and Health.

No. 1640, Vol. LXIII. SATURDAY, NOVEMBER 10, 1917. PRICE ONE PENNY

### MEDICINE IN THE GREAT WAR.

The middle way between opposing extremes is proverbially reported to be the safe way. On equal authority this desirable path is allowed to be both difficult to find and still more difficult to hold. An approximation to it is perhaps the best that average human nature may hope to attain, and even then, blown upon first from one side and then from the other, the course is apt to be zigzag rather than a steady line. If these propositions apply to judgments on questions of public policy in the smooth times of peace, they have a still more pressing application amidst the alternations of hope and depression which attend this tremendous day of war. The steady and relatively happy mean suffers much hindrance, and is in constant peril of deflection. Yet it may be that, especially in this acute moment, both our pessimists and our optimists have their values, for while the one without the other might permanently dislodge us, their combined efforts help towards a fair measure of stability. Though we may not go wholly with either, we may frankly acknowledge the merits of both. Unpopular with each, we are helped by them to a wide view and to a steady judgment. They are the advocates, and the plain undistinguished jury will wish to hear each of them before framing a verdict.

These remarks occur to us as we read the published accounts of the Chadwick Lecture on "The Part of Hygiene in the War" recently delivered by Dr. Woods Hutchinson. The lecturer bears a name which must ever have high honour in British medicine, and in his case the original stock has experienced the stimulating influence of a long residence in the Western world. Whether due to this environment or from other sources he has developed a breezy and forceful personality which shows itself in cheerful fashion in all he writes. In the United States he has become a kind of interpreter of medicine to the general public, and his books on various health topics are well known. He looks at facts through his own eyes and cultivates with success the saving grace of common sense. Recently he has visited the various fighting fronts of the European war, and his personal and professional equipment fit him admirably for a popular exposition of the medical organisations which are to be found there. If he seems to tell us experiences which are all comparatively pleasant reading we will not strive to repress or to qualify his ardour. He is at least "on the side of the angels," and so helps us to avoid the gloom that is apt to be engendered by voices of an opposite tendency. When we are told that the Navy is "rotten" and that the "hidden hand" moves as pawns those who are set in high places, it is a welcome word to learn on first-hand authority that at least in some directions we have proved equal to the occasion.

Especially in these columns are we bound to welcome a vindication of the medical services of our fighting forces. In our own way we have on more than one occasion attempted the task and have found that the facts make the enterprise an easy one. A new voice coming fresh from the scene of operations and speaking a confident message must therefore find here, as elsewhere, a sympathetic hearing. Dr. Hutchinson deals in facts and figures, and though some may feel that he gives to these a somewhat roseate tinge, the record can hardly be challenged. What medicine, alike in its preventive and in its curative aspects, has done for our armies, cannot be put in doubt, and the picture is made the more striking by contrast with former experiences. As part of the fighting forces the doctor has come into his own since it has been realised that, apart from his services to individuals, he is a large factor in keeping the ranks full and the army in being. The figures show that in former wars by far the larger part of the wastage of armies was caused by disease and not by wounds. Now the proportions are set in the opposite direction, and show something like sixteen deaths in battle to one from disease. It is meet and proper that scientific and practical medicine, even amidst the tragedy which still spreads loss and death in all our homes, should receive the meed of praise for this achievement. While, in the great phrase of John Bright, "the Angel of Death has been abroad in the land; you can almost hear the beating of his wings," the chapter would have been a still more poignant one but for the knowledge which, applied to practical ends, has banished epidemics and pestilences from our military camps and from our armies in the field. In similar fashion it is due to recognise, as the result of bacteriological investigations and surgical handicraft, the great saving of life and limb among those who fall wounded in the fight. It is said that 95 per cent. of those who reach the field hospitals recover from their injuries, and there is a message of relief in the announcement. By all means let us emphasise the fact, as it needs more than philosophy to see with composure the lame and stricken and blind who are in all our streets. It may be, as we are told, that the scalpel is mightier than the sword, and the test-tube than the trench-mortar. Our duty plainly is to remember these redeeming epigrams and by their aid to seek a right perspective, even though the sword and the trench-mortar and the poison gas take a daily and deadly toll of the fair promise of the nation. Such general and comprehensive propositions have their value and importance though they can do little to provide comfort for the lonely and bereaved. Indeed, to these such cheerful statistical calculations must seem an unfeeling exercise. To put the position in a word, it is that in the midst of a great human calamity medicine is doing something to lessen the horrors of the situation, and though inevitably the calamity suffers no cheerful talk, we must not forget the agencies which mitigate it.

